# Creating the Equivalence Index to Optimize the Precise Evaluation of Bee Products for Functionally Opposite Components

**DOI:** 10.3390/foods14091499

**Published:** 2025-04-25

**Authors:** Yongqing Wang, Feng Jia, Lu Zhang, Jingxian Jin, Pei Fan

**Affiliations:** College of Biological Engineering, Henan University of Technology, Zhengzhou 450001, China; wyq981303732@163.com (Y.W.); mrjiafeng@163.com (F.J.); zhanglu@haut.edu.cn (L.Z.); 231170400518@stu.haut.edu.cn (J.J.)

**Keywords:** Equivalence Index, bee products, functionally opposite components

## Abstract

Bee products, such as honey, bee pollen/bread, bee propolis and royal jelly foraged or secreted by honeybee workers, have been consumed by humans for many years and are important due to their complexity, the large number of them and the endemicity of their constituents. The health-promoting activities of bee products are widely documented all around the world. However, we have noticed a distinct but poorly described feature of bee products: groups of functionally opposite components (FOCs) related to blood sugar level, oxidative stress, cell membrane cholesterol distribution, cell membrane stability, cell membrane curvature, allergic reaction, cellular sodium influx and cardiac apoptosis that exist within these products. We then propose the Equivalence Index in order to overcome the challenges associated with FOCs; this is a concise mathematical model that can be used to optimize the evaluation of quality, determine any underlying mechanisms and provide processing guidance regarding bee products.

## 1. Introduction

Bees are an important insect and are kept and utilized worldwide. In addition to aiding in plant pollination, bees have provided food products for human consumption since ancient times. Natural bee products can be collective or secretive. Collective bee products include honey, bee pollen/bread (bee bread is the fermented form of bee pollen in honeycomb cells, while bee pollen is always collected at the entrance of the hive) and bee propolis, which are mainly sourced from plants with a small amount of bee secretion. Secretive bee products refer to royal jelly (secreted from the hypopharyngeal and mandibular glands of bee workers), bee venom (secreted from the venom gland) and beeswax (secreted from the wax gland). Among these products, bee venom and beeswax are always used as medical or industrial materials, rather than as food. Bee products are a cocktail of bio-active compounds. Honey and bee pollen contain approximately 200 components [1,2], while bee propolis contains more than 300 compounds [3]. These molecules comprise carbohydrates, proteins/peptides, lipids, vitamins and polyphenols, thus encompassing all the types of nutrients required to maintain human life. Hence, a distinguishing feature of bee products is the complexity and number of their components. Meanwhile, bee products are characterized by endemicity, with pronounced compositional discrepancies existing between the products produced by different bee species, plant resources, seasons and environments. Bee products could therefore be used as a good model food to investigate ingredient diversity.

Bee products, or the specific components in them, exhibit a broad range of health-promoting effects, including the modulation of microbial growth/infection, oxidation, inflammation, cancer, cardiovascular and neural systems [4]. Extensive research has been performed on the components and bio-activities of these bee products, with this research increasing year by year; this indicates that such traditional food remains attractive in the modern era. However, we have noticed that the different molecules within bee products may have opposing effects on human health. We call this a “dilemma of functionally opposite components (FOCs)”. This phenomenon is poorly described and discussed in the research. Therefore, we summarized the main FOCs in bee products based on the documented scientific data in order to highlight this issue and inspire novel research.

## 2. Blood Sugar Level: Glucose and Fructose vs. Oligosaccharides, Polysaccharides, Flavonoids and 10-Hydroxydec-2-Enoic Acid

Monosaccharides are prevalent in honey, bee pollen and royal jelly. The main monosaccharides present in bee products are fructose and glucose. In honey, monosaccharides have the highest content, accounting for approximately 70% of the total weight [1]. The fructose/glucose ratio can range from 1.3 to 1.6 [5]. In bee pollen, the contents of fructose and glucose can, respectively, amount to 21.44% and 17.40% in dry matter [6]. Bee propolis contains approximately 5% pollen [3]. Therefore, the contents of the two monosaccharides in bee propolis are comparatively low. In royal jelly, the content of fructose and glucose is 90% of the total sugars, with their concentrations ranging from 2.3% to 7.8% and 3.4% to 8.2%, respectively [7]. Fructose and glucose are important components of food as they provide energy for the metabolism of cells and can be used as a sweetener in food. However, the excessive intake of fructose and glucose may increase the risk of type 2 diabetes and is implicated in a range of metabolic diseases [8]. Therefore, the worldwide burden of hyperglycemia is an important health-related problem. As a food rich in fructose and glucose, bee products (particularly honey) have received significant attention with regard to glycemic control.

In fact, the glycemic index of natural honey is not very high, ranging from 32 to 85 based on the variety of honey [9]. The use of honey alone or synergically with other food can even lower blood sugar levels [10]. Many reports have suggested that oligosaccharides are the main components able to maintain a low glycemic level. The content of oligosaccharides such as maltose, sucrose, trehalose, isomaltosylglucose, pantose and erlose in honey, bee pollen and royal jelly is approximately 10% or lower [11,12]. Bee pollen also contains dietary fibers (polysaccharides) with a content ranging from 0.3 to 20 g/100 g of dry weight [13]. The content of soluble dietary fibers in bee pollen ranges between 4% and 9%, which is higher than that in many cereals and pulses [14]. Because the oligosaccharides in honey affect the gut microbiota, they may be the key constituents involved in altering the lipid metabolism of the host and overcoming diabetes [15]. The polysaccharides in bee pollen have been reported to promote the proliferation of pancreatic β-cells and insulin synthesis, thereby decreasing the content of blood glucose; this indicates that it plays a role in remedying type 1 diabetes [16]. Additionally, flavonoids, which ameliorate blood glucose levels [17], are the key polyphenols in honey (31.5–126.6 mg of gallic acid equivalents/100 g), bee pollen (1.00–5.50 mg of quercetin equivalents/g) and bee propolis (33–53 mg of quercetin equivalents/g) [12]. The fatty acid 10-hydroxydec-2-enoic acid (10-HDA), which exclusively presides in royal jelly with a concentration of 1.4–2.0% [18], is able to reduce blood glucose and to enhance the insulin levels of type 2 diabetic mice via the modulation of the PI3K/AKT/GSK3β signaling pathway [19]. The ability of monosaccharides and oligosaccharides, polysaccharides, flavonoids and fatty acids to modulate blood sugar levels is a distinctive characteristic of bee products.

## 3. Oxidative Stress: Glucose, Fructose, Glucose Oxidase and Omega-6 Fatty Acids vs. Vitamins, Flavonoids, Phenolic Acids, Omega-3 Fatty Acids, 10-HDA and Catalase

Glucose produces lipid hydroperoxides and thus promotes the formation of a cholesterol crystalline domain in the model membrane. Consequently, the width of the membrane bilayer decreases [20]. Such changes in the physicochemical properties of the membrane may result in oxidative stress-related pathogenesis. Furthermore, fructose has a stronger ability to induce the free radical peroxidation of natural lipid–protein supramolecular complexes than glucose [21]. These two monosaccharides may increase the oxidative harm posed by the lipid membrane. Honey contains glucose oxidase, which catalyzes glucose to glucose acid and hydrogen peroxide (H_2_O_2_); the latter compound is one of the main components that enables honey to kill microbes. As a member of ROS, H_2_O_2_ is capable of inducing cellular oxidative stress.

Bee pollen has a comparatively high content of lipids (1–13%). Linoleic acid is one of the major unsaturated fatty acids in bee pollen, with a content that can be higher than 10% of the total fatty acids [12,22,23]. Royal jelly also contains a small content of linoleic acid [24]. Linoleic acid and arachidonic acid, the lipid constituents of cell membranes, are omega-6 fatty acids. Linoleic acid is the precursor of arachidonic acid, and arachidonic acid is a precursor to a subset of pro-inflammatory mediators such as prostaglandins and leukotrienes. Therefore, arachidonic acid promotes inflammation in many cell types [25]. The high concentrations of arachidonic acid can induce oxidative stress in vivo and cause cell injury [26].

Bee products contain a range of anti-oxidant components, including vitamins, flavonoids, phenolic acids, catalase, and fatty acids like omega-3 fatty acids and 10-HDA. α-Tocopherol is an isoform of vitamin E, a representative vitamin in bee products, whose concentrations are 0.35 ng/g in honey [27], 16 μg/g in royal jelly and 80 μg/g in bee bread [28]; α-Tocopherol remedies carbon tetrachloride (CCl_4_)-induced lipid alterations, which materializes as against the increased phospholipid/protein ratio, sphingomyelin and phosphatidylcholine, but the decreased level of phosphatidylethanolamine in the membrane of liver cells. The protective effect of α-tocopherol may be associated with its anti-oxidant activity [29]. Flavonoids have anti-oxidant activities that are able to protect the lipid membrane. The incorporation of flavonoids into the cell membrane can easily occur. The hydrophobicity of flavonoids causes them to preferentially localize in the hydrophobic core of the membrane. With an increase in the number of hydroxyl groups attached to the backbone, their distribution tends to shift to the hydrophilic head [30]. The insertion of flavonoids, such as quercetin and myricetin, into the membrane can prevent oxidative attack and structural change [31]. Caffeic acid, a typical phenolic acid, also exhibits strong intracellular anti-oxidant capacities but a hydrophilic character. The introduction of alkyl chains into caffeic acid can promote its location within the membrane, thus conferring better protection against lipid peroxidation [32]. 10-HDA is a hydroxyl unsaturated fatty acid that can bind phosphatidylcholine and phosphatidylethanolamine (particularly the phosphatidylethanolamine), the major constituents of cell membrane phospholipids [33]. The hydroxyl group and -C=C- facilitate the scavenging of free radicals, indicating its potential ability to prevent oxidative damage being caused to lipids [18].

Eicosapentaenoic acid (EPA) and docosahexaenoic acid (DHA), which are also present in bee pollen [34], are omega-3 fatty acids. In contrast to linoleic acid and arachidonic acid, EPA and DHA are able to generate resolvins, which possess anti-oxidative stress and anti-inflammation properties [35,36,37]. The omega-3/omega-6 ratio varies significantly in different bee pollens; for example, in India, this ratio is from 0.06 to 3.09 [34].

Catalase, which breaks down H_2_O_2_ to water, also exists in honey. Therefore, the balance between glucose oxidase and catalase in honey determines the amount of H_2_O_2_, thus controlling the anti-microbial and anti-oxidant properties of honey. In a film-shaped polymer-based method, it was found that the activities of glucose oxidase and catalase were up to 1490.4 µg H_2_O_2_ ghoney^−1^ and 81.3 × 10^−3^ ghoney^−1^min^−1^, respectively, among 29 honey samples [38].

Cooperation among vitamins, flavonoids, phenolic acids, omega-3 fatty acids, 10-HDA and catalase could provide an effective approach to offsetting the oxidative damage caused by glucose, fructose and glucose oxidase in bee products.

## 4. Cell Membrane Cholesterol Distribution: EPA vs. DHA

Organisms can readily incorporate fatty acids from the environment in order to change the composition of the cell membrane, thus altering the properties of the membrane. EPA and DHA can be anchored by phospholipid molecules in the cell membrane [39]. Although EPA and DHA have a similar effect on resolving oxidative stress to the omega-3 fatty acids, they are different in terms of their chain length and the number of double bonds, resulting in EPA being extended but DHA bent in the membrane [40]. Correspondingly, EPA and DHA maintain and prevent the distribution of cholesterol, respectively. If the membrane possesses a high level of cholesterol, EPA and DHA have a distinct effect on the area expansion modulus (K_a_) of the membrane; this reflects the membrane strain, which controls the activity of the channel. The K_a_ is reduced by the inclusion of DHA, in comparison to that of EPA. This could be explained by the larger cholesterol crystalline nanodomains that are generated by DHA [41]. Another study also revealed that, without the 1-palmitoyl-2-oleoyl-sn-glycero-3-phosphocholine (POPC) and/or cholesterol, EPA and DHA have a similar *d*-space or intrabilayer distance. However, in the presence of POPC and cholesterol, EPA and DHA have contrasting effects; EPA and DHA, respectively, increase the electron density of the membrane hydrocarbon core and the electron density in the phospholipid head group region, but with hydrocarbon core disordering [42]. The difference in the effect of EPA and DHA on membrane cholesterol suggests that they could be associated with pathogenesis in different ways.

## 5. Cell Membrane Stability: Quercetin and Cyanidin vs. Naringenin, Chrysin and Unsaturated Fatty Acids

The flavonoids contained in honey, bee pollen or bee propolis, such as quercetin, cyanidin and their *O*-glucosides, can be inserted into the hydrophobic core of the membrane to form a channel-like structure due to their polyhydroxylated structure, lipophilicity and planar conformation [43]. Quercetin was shown in another study to increase the anisotropy and polarization of the cell membrane and to decrease its fluidity, therefore stabilizing the structure of the membrane [44].

Naringenin tends to insert itself into the hydrophobic layer, which is underneath the phospholipid heads of the erythrocyte membrane. This insertion leads to the order of molecules becoming disturbed and an increase in entropy, thus leading to an increase in the fluidity of the membrane [45]. Near the lipid–water interface, the chrysin–copper complex, much more than chrysin alone, exhibits a strong surface interaction and is partially inserted into the membrane bilayer, distorting the architecture of the lipid bilayer and increasing the flexibility of the membrane [46]. Although flavonoids have the ability to interact with the membrane, their different modes of interaction demonstrate that flavonoids have a comprehensive effect on cell functionality. Furthermore, many unsaturated fatty acids are capable of enhancing cell membrane fluidity [33,41]. The abnormally high or low fluidity of a cell membrane may produce different cell or body disorders. It is reasonable to precisely utilize these small molecules in ameliorating the cell membrane status.

## 6. Cell Membrane Curvature: Oleic Acid vs. Vitamin E

The flexibility of the cell membrane allows the membrane to curve, bending upwards or downwards. Oleic acid, a fatty acid present in bee pollen (even predominant in some samples) [23] and royal jelly [24], rapidly binds the lipid domain and crosses the membrane via the flip–flop mechanism, sometimes without the assistance of transport proteins [47]. The insertion of oleic acid, which has a cone shape, into the membrane causes positive curvature and a change in the organization and dynamics of the membrane; membrane fusion is therefore inhibited [48]. Membrane fusion enables enveloped viruses to enter the host cells. Supplying oleic acid via food might contribute to the inhibition of viral infection.

Besides reducing lipid peroxidation as is mentioned above, α-tocopherol is identified to change the cell membrane structure, hence to modulate the membranous behavior. In the model membrane, α-tocopherol contributes a spontaneous negative curvature with a radius of −13.7 Å, which might result from the inverted triangle-like insertion into the lipid membrane [49]. The bending of the membrane causes stress to the membrane proteins, thus changing their activities. Wedging into the membrane lipids in different modes makes oleic acid and α-tocopherol play contrasting roles in altering cell membrane curvature. Positive and negative curvatures may have different effects on the proteins, and this requires further investigation.

## 7. Allergic Reaction: Bee Pollen Allergens vs. Flavonoids

Bee pollen contains a large number of allergens that can cause anaphylaxis, making bee pollen dangerous in some cases [50]. Proteins such as calmodulins, profilin, expansins, pollen-specific proteins, major royal jelly proteins and Sal ks in bee pollen are considered key allergens [51].

Interestingly, the flavonoids present in bee pollen are associated with anti-allergic activities via the inhibition of interleukin-4 and interleukin-13 from T lymphocytes and IgE from B lymphocytes, the formation of the allergen–IgE complex and binding in mast cells or basophils [52]. This dual effect suggests that bee pollen has multiple uses with regard to allergy.

## 8. Cellular Sodium Influx: Grayanotoxins vs. Flavonoids

Honey contaminated by *Rhododendron* spp. nectar, which is referred to as the “mad honey”, contains grayanotoxins that threaten human health [53]. Although such honey is not commonly consumed worldwide, poisoning incidents have occurred in some regions [54,55], which attracted public attention. Grayanotoxins are able to induce neural, cardiovascular and muscular disorders [56], causing clinical symptoms such as bradycardia. One of the important mechanisms underlying the pathogenesis is the block of sodium inactivation via binding to the voltage-gated sodium channels in their open state [57]. Consequently, the sodium influx and depolarization could be prolonged.

Flavonoids are also characterized as the regulator of sodium channels but may have different modes from grayanotoxins. For instance, quercetin can inhibit voltage-gated sodium channels, thus reducing sodium influx in the cardiac system. Therefore, quercetin may have the anti-arrhythmic effects [58]. Similarly, genistein is identified to impede the sodium influx through the voltage-sensitive sodium channels in neurons [59]. The honeys have a high concentration of flavonoids which might partly counteract the intoxication of the grayanotoxins.

## 9. Cardiac Apoptosis: Grayanotoxins vs. 10-HDA

As is described in the previous section, the heart is a key target of grayanotoxins. Experiments in vivo proved that cardiac apoptosis increases with the administration of grayanotoxin-III, indicating the apoptotic induction may also be an important mode for the grayanotoxin toxicities [60]. A recent study revealed that pre-treatment with 10-HDA attenuates cardiac apoptosis and enhances cardiac function in the model of myocardial ischemia/reperfusion injury [61]. The data suggest that 10-HDA originating from royal jelly could protect heart health against grayanotoxins in the perspective of apoptosis. The induction/reduction of apoptosis might be dependent on cell types; the effects of grayanotoxins/10-HDA on the apoptosis of other cells still need further investigation.

Unlike the grayanotoxins naturally existing in “mad honey”, 5-hydroxymethylfurfural (5-HMF) might occur during the honey processing and storage stages [62]. However, the effect of 5-HMF remains controversial. 5-HMF could be a member of the FOCs if its effect is determined in the future.

**Figure 1:**
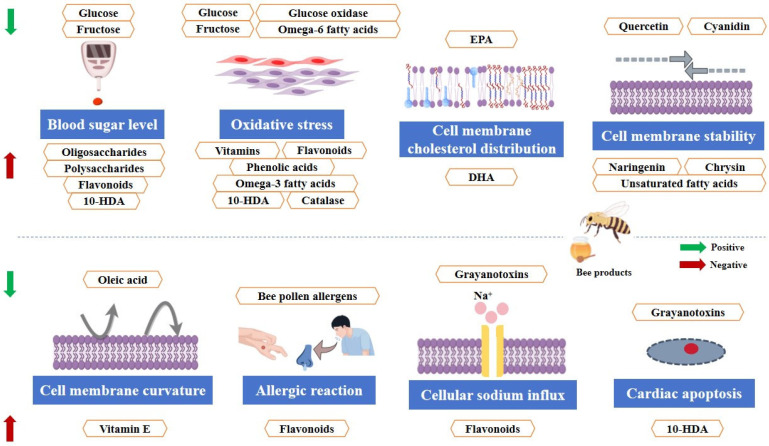
The major FOCs in bee products (created by Figdraw).

A pictorial representation of the FOCs present in bee products is shown in Figure 1. However, with an in-depth elucidation of the functions of the molecules present in bee products, these groups of FOCs may be enlarged.

## 10. Equivalence Index for FOCs in Bee Products for the Precise Evaluation of Quality and Other Niches

Although the two counterparts in a group of FOCs exert explicit roles, the ability of quantitative analysis to create their equivalence for an effect is currently unknown. Here, we proposed the Equivalence Index (*EI*), which is shown in Formula (1), to represent the ratio of the two counterparts in a group of FOCs when the effect attains equivalence.

(1) $${EI}_{effect}=\frac{a(X)}{a^{\prime }(Y)}$$

In the formula, *a*(*X*) and *a’*(*Y*) are the amounts of *X* and *Y* used, respectively, when the effect is neutralized. Further, based on the real ratio (*RR*) of the two counterparts in a bee product, shown as *A*(*X*) and *A’*(*Y*), respectively (2), we created the Quality Index (*QI*); this was in order to symbolize the quality of a bee product in terms of the targeted effect (3).

(2) $$RR=\frac{A(X)}{A^{\prime }(Y)}$$

(3) $${QI}_{effect}=\frac{{EI}_{effect}}{RR}=\frac{a(X)A^{\prime }(Y)}{a^{\prime }(Y)A(X)}$$

If *X* and *Y* are single molecules, *EI* can be shared when the batches of a bee product are compared for a certain effect. If, and in most cases, the two counterparts both contain multiple molecules, the situation would be more complex due to the different combinations of molecules producing various effect values. For instance, the *EI_bloodsugar_* of honey (*Δ*) or honey (*Ω*) in terms of monosaccharides vs. oligosaccharides and flavonoids is expressed as (4) or (5). Because the types and quantities of monosaccharides, oligosaccharides and flavonoids may be varied in the two honeys, *a*(*M*) and *a’*(*O* + *F*) refer to the quantity of monosaccharides and oligosaccharides + flavonoids in honey (*Δ*), while *a*(M*) and *a’*(O* + F*) denote that in honey (*Ω*). *M* and M*, as well as *O* + *F* and O* + F*, represent the various sub-constituents in different honeys.

(4) $${EI}_{(\Delta )bloodsugar}=\frac{a_{\left(\Delta \right)}(M)}{{a^{\prime }}_{\left(\Delta \right)}(O+F)}$$

(5) $${EI}_{(\Omega )bloodsugar}=\frac{a_{\left(\Omega \right)}(M*)}{{a^{\prime }}_{\left(\Omega \right)}(O*+F*)}$$

*EI_bloodsugar_* compared to _the_ *RR* of monosaccharides vs. oligosaccharides + flavonoids, shown as *QI_bloodsugar_* of honey (*Δ*) (6) and honey (*Ω*) (7), respectively, could be used to reflect the ability of the two honeys to regulate blood sugar levels.

(6) $${QI}_{(\Delta )bloodsugar}=\frac{{EI}_{(\Delta )bloodsugar}}{{RR}_{\left(\Delta \right)}}=\frac{a_{\left(\Delta \right)}(M){A^{\prime }}_{\left(\Delta \right)}(O+F)}{{a^{\prime }}_{\left(\Delta \right)}(O+F)A_{\left(\Delta \right)}(M)}$$

(7) $${QI}_{(\Omega )bloodsugar}=\frac{{EI}_{(\Omega )bloodsugar}}{{RR}_{\left(\Omega \right)}}=\frac{a_{\left(\Omega \right)}(M*){A^{\prime }}_{\left(\Omega \right)}(O*+F*)}{{a^{\prime }}_{\left(\Omega \right)}(O*+F*)A_{\left(\Omega \right)}(M*)}$$

If (6) > (7), the inhibitory effect that honey (*Δ*) has on blood sugar levels would be better than that of honey (*Ω*) for the larger proportion of oligosaccharides + flavonoids and vice versa. Therefore, *EI* can be used to precisely evaluate the quality of a bee product for a specific purpose. Future work is required to determine the *RRs*, *EIs* and *QIs* for the FOCs of the bee products in order to create *EI* systems.

To determine *EI* and *QI* includes three key steps, which are featured by “Composition–Function–Evaluation”:

Step 1: To specify and to quantify the FOCs for a particular purpose in one type of the bee products. The qualitative and quantitative methodologies to detect a series of FOCs of bee products (monosaccharides, oligosaccharides, polysaccharides, flavonoids, fatty acids, vitamins and proteins) include the widely used chromatography, mass spectrometry, Raman spectrometry or their integration [63,64,65,66,67,68,69,70,71,72]. Accordingly, the *RR* can be calculated. For better utilization of *EI* and *QI*, a group of FOCs with more than two molecules should be profiled (the specific types of the molecules of the FOCs and their amounts are needed). The different combinations of the molecules may produce effects with varying extents.

Step 2: To compare the strengths of the increasing side and the decreasing side of the FOCs of interest; that is, to analyze what amount of *Y* is able to neutralize the effect of the setting amount of *X*. The parameters and processes for analyzing blood sugar, oxidative stress, cell membrane cholesterol distribution, cell membrane stability, cell membrane curvature, allergic reaction, cellular sodium influx and cardiac apoptosis are implicated in the previously cited literature [16,20,41,42,45,48,49,52,58,60]. Therefore, the *EI* can be determined.

Step 3: To calculate the *QI* that is based on *RR* and *EI*, which can be used to evaluate the bee product quality for a targeted effect.

Currently, the reported works let us know the varieties and the amounts of the components in bee products, which enables us to calculate *RRs* in many cases. However, the interactions of the two counterparts from FOCs are rarely known. Hence, we are unable to calculate *EIs* using the present data. This is an important reason why we believe the determination of *EIs* is novel and crucial. Therefore, we use the hypothetical data to make the concept clear.

Here is a case to calculate *RRs* for two honey samples (raw green honey and Tualang honey) according to the published data [73]. Raw green honey and Tualang honey are marked as (*Δ*) and (*Ω*), respectively. Honey (*Δ*) contains 23.7 g/100 g glucose and 11.35 g/100 g fructose, while honey (*Ω*) contains 28.79 g/100 g glucose and 31.73 g/100 g fructose. Then, *M* and M* are 35.05 g/100 g and 60.52 g/100 g for honey (*Δ*) and honey (*Ω*). The total sugar contents in honey (*Δ*) and honey (*Ω*) are, respectively, 39.3 g/100 g and 63.4 g/100 g. Honey sugar is basically composed of monosaccharides and oligosaccharides. Therefore, the oligosaccharide content is shown as the total sugar content subtracting monosaccharide (glucose and fructose) content. The amounts of total flavonoids in honey (*Δ*) and honey (*Ω*) are 0.0076 g/100 g and 0.0405 g/100 g, respectively. The *O* + *F* for honey (*Δ*) is 4.2576 g/100 g and O* + F* for honey (*Ω*) is 2.9205 g/100 g. The *RR_(Δ)_* is 8.2323 while the *RR_(Ω)_* is 20.7225. Considering the compositional variations between *M* and M*, as well as *O* + *F* and O* + F*, the *EI_(Δ)bloodsugar_* and *EI_(Ω)bloodsugar_* have to be calculated independently. As is described above, no data are available to calculate the *EIs*; the hypothesized values will be used as follows. For honey sample (*Δ*), if the oral administration of 1 g *M* increases 1 mM blood sugar in mice, while 2 g *O* + *F* reduces that 1 mM, the *EI_(Δ)bloodsugar_* is 0.5. For honey sample (*Ω*), if the oral administration of 0.8 g M* increases 1 mM blood sugar in mice, while 2.2 g O* + F* reduces that 1 mM, the *EI_(Ω)bloodsugar_* is 0.36. An *EI* value is indicative of the antagonistic degree of the two counterparts from the FOCs in a sample. The potentially different *EI* values would symbolize the functional discrepancy between the two samples, which could result from their compositional variations. Moreover, *QI_(Δ)bloodsugar_* and *QI_(Ω)bloodsugar_* are 0.061 and 0.017, respectively, which indicates that the intake of honey (*Δ*) may have a better effect on blood sugar control. But, for hypoglycemia, honey (*Ω*) could be more suitable for dietary supply. In addition to intuitively displaying the divergence for an effect between samples, *EI* helps to understand the relationships between the sub-component features and the grades of their activities.

*EI* is a concise mathematical model that can be used to link the components of bee products with their relevant functions when the functional oppositions achieve balance. With the introduction of *EI*, the quality of bee products can be evaluated precisely from the perspective of a targeted function. The preferred function of a bee product could also be predicted based on the *EI*. Such benefits would lead to the optimized consumption of bee products, thus promoting the global trade in bee products. On the other hand, *EI* will enhance our understanding and mechanistic disclosure of the complex components that influence the bio-activities of bee products.

The *EI* could also be applied to food processing guidance, with the apiculture and bee product industry making use of the *EI*. Bee products are primarily processed via the mixing of different bee products to create food, which has been performed for many years. Honey is traditionally added to bee pollen and royal jelly to enhance their taste. Currently, such mixtures provide enhanced ingredients and bio-activities [74,75,76]. Moreover, bee products are often used to create new forms of food, such as beverages, alcoholic drinks, snacks and dietary supplements, that are commercially available. In these processes, the *EI* of FOCs could be used to create better recipes and enhance the use of nutritional properties.

In conclusion, using *EI* is a feasible approach to overcoming the challenges associated with FOCs in both basic research and the industrial application of bee products. Our next work is to experimentally determine the *EIs*, which is urgent. However, we need to notice that *EIs* should be dynamic. With the new findings on bee product components, *EIs* could be updated.

## Data Availability

No new data were created or analyzed in this study. Data sharing is not applicable to this article.

## Abbreviations

The following abbreviations are used in this manuscript:

FOCs	functionally opposite components
10-HDA	10-hydroxydec-2-enoic acid
CCl_4_	carbon tetrachloride
H_2_O_2_	hydrogen peroxide
EPA	eicosapentaenoic acid
DHA	docosahexaenoic acid
POPC	1-palmitoyl-2-oleoyl-sn-glycero-3-phosphocholine
5-HMF	5-hydroxymethylfurfural
*EI*	Equivalence Index
*RR*	real ratio
*QI*	Quality Index
